# Construction of an Immunosensor Based on the Affinity DNA Functional Ligands to the Fc Segment of IgG Antibody

**DOI:** 10.3390/bios15110747

**Published:** 2025-11-05

**Authors:** Qianyu Yang, Zhiwei Liu, Xinrui Xu, Zihao Zhao, Ze Fan, Bin Du, Jianjie Xu, Jiwei Xu, Jiang Wang, Bing Liu, Xihui Mu, Zhaoyang Tong

**Affiliations:** State Key Laboratory of Chemistry for NBC Hazards Protection, Beijing 102205, China; qyyang918@163.com (Q.Y.); xuxinrui@163.com (X.X.); zzhqfnu1012@163.com (Z.Z.); fzhnu@hnu.edu.cn (Z.F.); dubin51979@163.com (B.D.); xujianjie@sklnbcpc.cn (J.X.); xujw14@mail.ustc.edu.cn (J.X.); roverman@163.com (J.W.); lbfhyjy@sohu.com (B.L.); muxh0511@163.com (X.M.)

**Keywords:** DNA functional ligand, Fc fragment, directional immobilization, toxin detection, immunosensor

## Abstract

Over the past few decades, Fc fragment-conjugated proteins, such as Protein A, have been extensively utilized across a range of applications, including antibody purification, site-specific immobilization of antibodies, and the development of biosensing platforms. In this study, building upon our group prior research, we designed and screened an affinity DNA functional ligand (A-DNAFL) and experimentally validated its binding affinity (*K_D_* = 6.59 × 10^−8^) toward mouse IgG antibodies, whose binding performance was comparable to that of protein A. Systematic evaluations were performed to assess the binding efficiency under varying pH levels and ionic strength conditions. Optimal antibody immobilization was achieved in PBST-B buffer under physiological pH 7.2–7.4 and containing approximately 154 mM Na^+^ and 4 mM K^+^. Two competitive binding assays confirmed that the A-DNAFL binds to the Fc fragment of murine IgG antibody. Furthermore, molecular docking simulations were employed to investigate the interaction mode, revealing key residues involved in binding as well as the contributions of hydrogen bonding and hydrophobic interactions to complex stabilization. Leveraging these insights, A-DNAFL was utilized as a tool for oriented immobilization of antibodies on the sensing interface, enabling the construction of an immunosensor for ricin detection. Following optimization of immobilization parameters, the biosensor exhibited a detection limit of 30.5 ng/mL with the linear regression equation is lg(*Response*) = 0.329 lg(*C_ricin_*) − 2.027 (*N* = 9, *R* = 0.938, *p* < 0.001)—representing a 64-fold improvement compared to conventional protein A-based methods. The system demonstrated robust resistance to nonspecific interference. Sensing interface reusability was also evaluated, showing only 8.55% signal reduction after two regeneration cycles, indicating that glycine effectively elutes bound antibodies while preserving sensor activity. In summary, the A-DNAFL presented in this study represents a novel antibody-directed immobilization material that serves as a promising alternative to protein A. It offers several advantages, including high modifiability, low production cost, and a relatively small molecular weight. These features collectively contribute to its broad application potential in biosensing, antibody purification, and other areas of life science research.

## 1. Introduction

Antibodies are Y-shaped proteins produced and secreted by plasma cells, which are essential components of the biological immune system, utilized for the recognition and neutralization of foreign substances such as bacteria and viruses. Antibodies play a crucial role in various fields including life sciences, medicine, and environmental chemistry, and have been widely applied in disease diagnosis and therapy [1,2,3,4,5,6], vaccine evaluation [7,8,9], and biochemical molecular detection [10,11,12,13,14]. A complete antibody molecule consists of three functional regions: two identical antigen-binding fragments (Fab) and one crystallizable fragment (Fc). Each Fab contains an antigen-binding site that specifically recognizes and binds to a particular target antigen [15]. In contrast, the glycosylated Fc region interacts with various receptor molecules that are involved in modulating and executing antibody-mediated immune responses [16,17,18,19].

Protein A, isolated from the cell wall of Staphylococcus aureus type A, is well recognized for its specific binding affinity to the Fc region of immunoglobulins [20,21]. Subsequently, additional antibody-binding proteins, including protein G and protein L, have been identified and utilized [22,23]. To date, these proteins have become essential tools in biomedical research and industrial applications, with their use extending beyond traditional antibody purification [24,25,26,27,28] to areas such as immunodetection [29,30], antibody engineering [31,32], and pharmaceutical development [33,34,35]. The discovery of protein A has significantly advanced the efficient production and application of antibodies. Although comprehensive data on the direct economic impact of protein A is not publicly available, a report by Research and Markets indicates that the global market for protein purification reagents reached USD 23.65 billion in 2023, which underscores the substantial commercial value of protein A and related materials. Currently, the most significant economic contribution of protein A lies in the manufacturing of monoclonal antibodies (mAbs) [36,37,38]. Despite the considerable progress achieved, challenges remain due to its high molecular weight, elevated production costs, complex purification procedures, limited stability under alkaline conditions, and stringent elution requirements [39,40,41,42,43]. Consequently, the development of novel Fc-binding agents that offer comparable functionality but with lower molecular weight and reduced production costs is of considerable scientific and industrial importance.

To achieve this objective, it is important to evaluate the binding interaction between the novel immobilization materials and the Fc segment through appropriate methodologies. Commonly employed techniques include surface plasmon resonance [44,45,46,47], isothermal titration calorimetry [48,49,50,51] and fluorescence resonance energy transfer [52,53,54,55], all of which are well-established methods for assessing binding affinities and analyzing intermolecular interaction. Additionally, Bio-Layer Interferometry (BLI), a label-free, real-time biomolecular interaction analysis method based on light interference principles, eliminates the need for fluorescent or radioactive labeling. It is widely applied in the study of drug molecule binding kinetics, vaccine development, and antigen–antibody affinity determination [56,57,58,59,60,61]. When light propagates through the functionalized film layer at the tip of the fiber optic biosensor, the binding of biomolecules causes an increase in the film’s thickness. The light interference occurs between the reflected light waves generated at different locations, resulting in distinct interference spectra. By continuously monitoring the real-time shifts in these interference spectra, the dynamic parameters of molecular binding and dissociation processes can be quantitatively analyzed [62,63,64].

In preliminary studies conducted by our group, a series of DNA functional ligands (DNAFL) capable of binding to the Fc fragment of antibodies were identified [65]. Through virtual screening and experimental validation, a single-stranded DNA sequence was optimized and identified. Its binding affinity toward classical mouse IgG antibody was evaluated, which was named as A-DNAFL. Subsequently, the influence of varying pH and ionic strengths on the immobilization efficiency of A-DNAFL with the antibody was systematically investigated. The results of the competitive binding assay confirmed that the interaction sites were located within the Fc fragments. Molecular docking analyses were further conducted to elucidate potential binding surface and underlying molecular interaction mechanisms. Thereafter, A-DNAFL was employed as a recognition element for directional fixation of murine antibodies at the immune sensing interface, enabling the construction of a biosensor based on BLI for the detection of ricin. Notably, the biosensing interface retained antigen-binding activity even after multiple regeneration cycles. Therefore, the Fc-targeting A-DNAFL identified in this study holds promise as a novel alternative to protein A for oriented antibody immobilization, with broad potential applications in emerging biosensing platforms and life science research.

## 2. Materials and Methods

### 2.1. Reagents and Apparatus

DNAFL and biotinylated A-DNAFL were synthesized by Beijing Xing Fangyuan Biotechnology Co., Ltd. (Beijing, China). Ricin was purchased from Beijing Hapten and Protein Biomedical Institute (Beijing, China). Mouse anti-ricin monoclonal antibody (ricin McAb) was purchased from Hytest (Shanghai, China). ChromPure™ mouse IgG (whole molecule) was purchased from Jackson ImmunoResearch Inc (West Grove, PA, USA). Protein A, bovine serum albumin (BSA), phosphate-buffered saline (PBS) at various pH values (5.0, 6.0, 7.0, 7.2–7.4, 8.0, and 9.0), and PBS containing tenfold concentrations of potassium (K^+^) and sodium (Na^+^) ions were all procured from Solarbio (Beijing, China). Magnesium chloride (MgCl_2_), and ovalbumin were obtained from Macklin (Beijing, China). Human immunoglobulin G (human IgG) was purchased from Bioss (Beijing, China). Glycine was purchased from Innochem (Beijing, China). Tween 20 was purchased from Sigma-Aladdin (Shanghai, China). PBST-B solution was prepared using 0.01 M PBS that containing 0.02% Tween 20 and 0.5% BSA. PBST-glycine (pH 1.70, 0.01 M, containing 0.02% Tween 20 and 10 mM glycine) were prepared with deionized water. All other reagents were of analytical reagent grade and used without further purification.

Affinity was determined by Octec K2 Molecular Interactor by ForteBio (Fremont, CA, USA), and all steps were performed at 30 °C with shaking at 1000 rpm in a 96-well plate containing 200 µL of solution in each well. BLI sensors were purchased from Sartorius (Göttingen, Germany), and the types of sensors utilized in this research encompass High Precision Streptavidin (sensor type: SAX2.0) and Protein A (sensor type: ProA). The classical secondary structure and three-dimensional (3D) structure of DNAFL were predicted using RNAstructure and the XIAOLAB web server (http://biophy.hust.edu.cn/new/3dRNA (accessed on 10 April 2025)), respectively. Molecular docking simulations (MDs) were performed using Discovery Studio 4.5 software (DS 4.5).

### 2.2. Characterization of Affinity and Binding Sites

In our prior laboratory investigations, a series of DNA functional ligands (DNAFL) demonstrated binding affinity toward mouse IgG antibodies were identified. Through iterative screening, we successfully optimized and isolated DNA sequences exhibiting enhanced affinity. One high-performing sequence (5′-TCGCAAGACGGACAGAAGGCTTGTGGTCTTCTTTGGTGATGTACTGCCGTTAATGGAGTGTTGGTGGAGCGATTTGT-3′) that was selected and designated as A-DNAFL for further investigation in the present study.

#### 2.2.1. Characterization of Affinity

**Figure 1:**
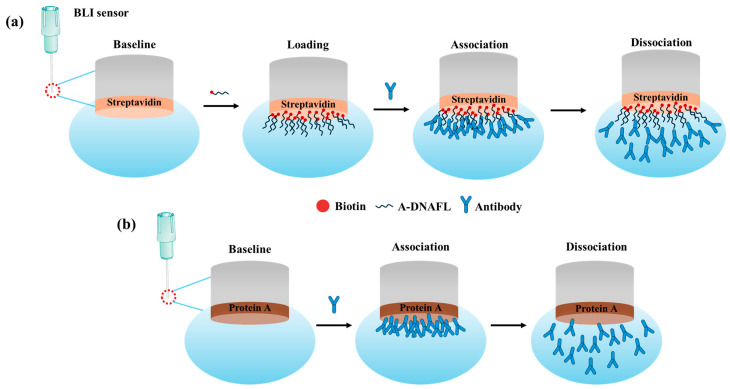
(**a**) Mechanism of the interaction between A-DNAFL and mouse IgG antibodies; (**b**) interaction between protein A and mouse IgG antibodies.

To determine the binding affinity between A-DNAFL and mouse IgG antibodies, the equilibrium dissociation constant (*K_D_*) was measured using the Octet K2 molecular interaction analysis system. A lower *K_D_* indicates a higher binding affinity. The assay was performed using SAX2.0 sensors, which were first equilibrated in PBST-B (pH 7.2–7.4) for 120 s to establish a baseline and activate streptavidin. Subsequently, the sensors were incubated with 0.5 μM biotinylated A-DNAFL for 600 s to loading. Following immobilization, the sensors were exposed to five serial dilutions of mouse IgG antibody solutions (0.0125, 0.025, 0.05, 0.1, and 0.2 mg/mL) for a 300 s association phase. This was followed by a dissociation process in PBST-B (pH 7.2–7.4), which also served as the reference solution. After each cycle, the sensors were regenerated using PBST-glycine to ensure complete removal of bound antibodies. For comparative purposes, the interaction between protein A and IgG antibodies was similarly evaluated. The detection principles are illustrated in Figure 1.

It is essential to systematically evaluate the influence of varying pH environments and ionic strength conditions on the performance of A-DNAFL’s immobilization efficiency to antibodies. Specifically, the pH of the phosphate-buffered saline in the PBST-B buffer solution was adjusted to 5.0, 6.0, 7.0, 8.0, and 9.0, respectively, and affinity measurements were conducted under each condition. Subsequently, under fixed optimal pH conditions, the binding affinity of A-DNAFL to the antibody was evaluated under varying ionic strength conditions, including a tenfold increase in K^+^ and Na^+^ ion concentrations in PBS, as well as the addition of 0.1 mM, 1 mM, and 10 mM MgCl_2_ (as mentioned in the literature [66]) to assess the influence of divalent cations.

#### 2.2.2. Verification of Binding Sites

To further confirm that the binding site of A-DNAFL on the IgG antibody is located within the Fc fragment, two kinds of competitive binding experiments between protein A and A-DNAFL were conducted. A schematic diagram is presented in Figure S1. In the first experimental setup, IgG antibody (0.2 mg/mL) was immobilized on the ProA sensor over a 10 min period, after which the sensor was transferred to a 96-well plate to undergo binding and dissociation reactions with A-DNAFL (0.5 μM), while maintaining all other conditions constantly. In an alternative approach, biotinylated DNAFL (0.5 μM) was initially immobilized on the SAX2.0 sensor surface who was subsequently transferred to the IgG (0.2 mg/mL) to allow for binding. Finally, the antibody-bound SAX2.0 sensor was exposed to 1 μM protein A solution for “association–dissociation” analysis. Negative control consisted of PBST-B under identical conditions.

#### 2.2.3. Molecular Docking Simulation Between A-DNAFL and Fc Fragment

3D crystal structure of mouse IgG was retrieved from the Protein Data Bank (PDB ID: 1IGT) and subsequently imported into DS 4.5 software to serve as the receptor molecule for MDs. Concurrently, the 3D structure of the A-DNAFL was predicted using the XIAOLAB web server and utilized as ligands in the MDs. Molecular docking was performed using the ZDOCK module within DS 4.5, and the parameter settings, evaluation of docking results, and analysis of binding interfaces were carried out following the protocols previously established in our laboratory [65].

### 2.3. Construction of Immunosensors

The A-DNAFL was employed as a novel tool for the direct immobilization of ricin McAb, and leveraging BLI technology, a new immunosensor was developed and applied for the detection of ricin.

Firstly, various concentrations (0.5, 1.0, 1.5, 2.0, 2.5, 3.0 and 3.5 μM) of biotinylated A-DNAFL was immobilized on the SAX2.0 sensor surface until signal saturation was achieved, to determine the optimal immobilization concentration of the A-DNAFL. Similarly, different McAb concentrations (0.1, 0.2, 0.3, 0.4, 0.5, 0.6 and 0.7 mg/mL) were applied to A-DNAFL to identify the optimal antibody immobilization level. Detection procedure of the developed immunosensor is illustrated in Figure S2. Following the sequential modification of the SAX2.0 sensor with DNAFL and ricin McAb, the functionalized sensor was exposed to samples containing varying concentrations of ricin for a 5 min binding period, during which the corresponding response signals were recorded, using PBST-B as the negative control Furthermore, the sensor’s interference resistance was assessed using two potential interfering substances (ovalbumin and human immunoglobulin G), and each ricin concentration measured three times to evaluate repeatability. The detection limit was determined, and the corresponding response curve was generated. For comparative purposes, the conventional protein A-based immobilization method for antibody attachment, applied in ricin detection, was also employed. The underlying principle is also illustrated in Figure S2.

Furthermore, this study investigated the reusability of the novel A-DNAFL-based sensor. A-DNAFL-modified SAX2.0 sensor was sequentially exposed to antibodies and antigens, then regenerated using a PBST-glycine treatment, after which it was promptly reused for another round of antibody–antigen binding. This regeneration and reuse cycle was repeated three times to evaluate the consistency of antigen recognition signals before and after regeneration. For comparison, the regeneration performance of the conventional protein A-based sensor was assessed under identical conditions.

## 3. Results

### 3.1. Interaction Between ssDNA and Mouse IgG

#### 3.1.1. Affinity

**Figure 2:**
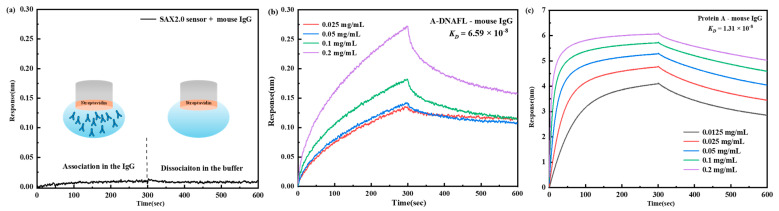
(**a**) Signal of the SAX2.0 sensor immobilizing IgG antibody (region to the left of the dashed line corresponds to the combination process, whereas the region to the right denotes the dissociation process.); (**b**) association–dissociation curve between A-DNAFL and mouse IgG antibody; (**c**) association–dissociation curve between protein A and mouse IgG antibody.

Based on prior relevant experimental findings in the laboratory, A-DNAFL, exhibiting affinity for mouse IgG antibodies, was successfully identified. Subsequently, the binding mechanism between this DNA sequence and mouse IgG antibodies was systematically investigated. Figure 2a illustrates the binding behavior of the SAX2.0 sensor with the IgG antibody, where no signal was observed, effectively eliminating the possibility of streptavidin interfering with antibody binding. Figure 2b displays the association and dissociation profiles of the A-DNAFL to the IgG antibody. When antibodies of varying concentrations bind to the BLI sensor, the thickness of the sensing layer increases, leading to changes in the optical interference signal. Any anomalous signals observed across the five binding datasets (e.g., the curve corresponding to the 0.0125 mg/mL in Figure 2b) were excluded from analysis; however, at least three valid data curves were retained to ensure reliable fitting of the *K_D_* value. The calculation formula for *K_D_* is presented in Equation (1), where *K_on_* denotes the association rate constant with units of 1/(M·s), and *K_dis_* denotes the dissociation rate constant with units of 1/s.

(1) $$K_{D}=\frac{K_{dis}}{K_{on}}$$

Therefore, the final fitted values for the *K_on_*, *K_dis_*, and *K_D_* of the A-DNAFL to IgG antibody are 1.74 × 10^4^, 1.15 × 10^−3^ and 6.59 × 10^−8^, respectively. In addition, Figure 2c presents the association–dissociation curves of protein A and the IgG, whose *K_on_*, *K_dis_*, and *K_D_* are 9.25 × 10^4^, 1.21 × 10^−3^ and 1.31 × 10^−8^, respectively. While based on the curve, the *K_D_* between protein A and the IgG is approximately five times higher than that of A-DNAFL.

Prior to constructing the immunosensor for toxin detection, the stability of the immobilization system as well as its applicable environmental parameters was assessed. For clarity, the affinity constant (*K_A_*) is introduced, defined as the reciprocal of the equilibrium dissociation constant (*K_D_*), as expressed in Equation (2). Based on this calculation, the *K_A_* value of A-DNAFL reported in this study is 1.52 × 10^7^, which provides a more intuitive indication of binding strength.

(2) $$K_{A}=\frac{1}{K_{D}}$$

**Figure 3:**
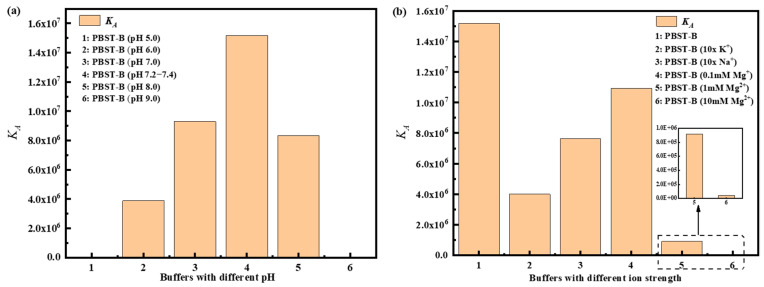
Affinity of A-DNAFL and IgG antibodies in (**a**) different pH environments and (**b**) different ionic strength buffer systems.

PBST-B was prepared using five distinct pH of PBS buffer, and the binding affinities of A-DNAFL with IgG antibodies under these conditions are presented in Figure 3a, corresponding to samples 1, 2, 3, 5, and 6, whereas sample 4 served as a reference control representing pH 7.2–7.4. Within the pH range of 6.0 to 8.0, maximum affinity was observed at pH 7.2–7.4, where the antibody’s net charge approached neutrality, resulting in minimal electrostatic interaction with the negatively charged DNA framework and thereby reducing non-specific binding. At pH 5.0, the antibody exhibited a substantial positive charge, leading to strong classical adsorption onto nucleotides. When the SAX2.0 sensor functionalized with biotinylated A-DNAFL was immersed in the antibody solution, rapid adsorption of the antibody onto the sensor surface occurred, yielding a pronounced signal enhancement. However, binding signals across five concentrations showed negligible variation, lacking a discernible gradient (see Figure S3a). Furthermore, the association–dissociation profile obtained at pH 9.0 was anomalous (see Figure S3e), as the response signals failed to exhibit reliable concentration dependence. Consequently, accurate *K_D_* values could not be determined through curve fitting under these two conditions. In the case of pH 9.0, no low-response phase indicative of electrostatic repulsion was detected, likely due to alterations in molecular physicochemical properties, less predictable charge interactions, and increased dominance of hydrophobic forces, which may promote non-specific hydrophobic binding.

Based on the optimal pH range of 7.2–7.4, we further examined the affinity under varying ionic strengths, as illustrated in Figure 3b. Initially, the concentrations of both monovalent cations in PBS increased tenfold, yielding approximate concentrations of 40 mM for K^+^ and 1.54 M for Na^+^. Compared to standard conditions, the *K_D_* significantly decreased, indicating that elevated ionic strength shields the surface charges of A-DNAFL and antibody, thereby weakening binding interactions and accelerating dissociation. Furthermore, the curves presented in Figure S4a,b were fitted to derive effective *K_D_*, suggesting that a moderate increase in these ion concentrations does not induce complete denaturation or functional loss of either the antibody or A-DNAFL. Upon addition of 0.1 mM MgCl_2_, the *K_D_* was slightly reduced compared to standard PBST-B conditions. When the Mg^2+^ ion concentration was raised to 1 mM, the affinity declined by an order of magnitude, likely due to enhanced electrostatic shielding. At 10 mM Mg^2+^, the *K_D_* value decreased by another order of magnitude, reaching 10^4^ M^−1^, and exhibited unstable binding behavior (Figure S4d). In summary, excessive concentrations of K^+^ and Na^+^, as well as increasing Mg^2+^ levels, disrupt the electrostatic interactions between A-DNAFL and IgG antibodies, resulting in diminished affinity. Therefore, conventional PBST-B buffer (pH 7.2–7.4, containing approximately 154 mM Na^+^ and 4 mM K^+^) remains the most suitable environment for immunosensor development. (Association–dissociation curves for all samples in Figure 3 are provided in Figures S3 and S4).

#### 3.1.2. Validation Binding Sites

**Figure 4:**
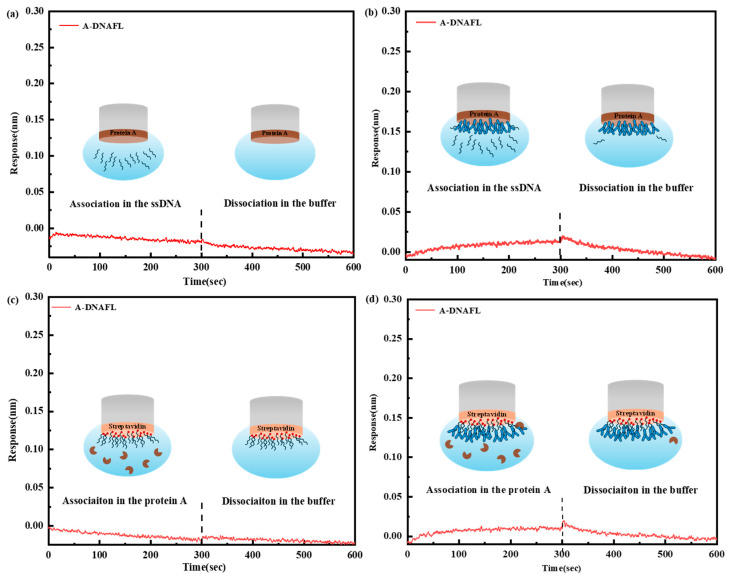
(**a**) Response of ProA sensor to A-DNAFL; (**b**) competition between A-DNAFL and the antibodies fixed on the ProA sensor; (**c**) response of the A-DNAFL fixed SAX2.0 sensor to protein A; (**d**) competition between protein A and IgG antibodies fixed by A-DNAFL (region to the left of the dashed line corresponds to the combination process, whereas the region to the right denotes the dissociation process).

In the control experiment presented in Figure 4a, the ProA sensor was directly introduced into various A-DNAFL solutions. No significant response signal was detected, indicating the absence of specific adsorption between protein A and the A-DNAFL. Subsequently, when the IgG antibody was immobilized onto the ProA sensor and exposed to the same A-DNAFL solutions, only minimal binding was observed. This weak interaction may be attributed to a small amount of DNA adsorption onto the Fc segment, which was not entirely shielded by protein A due to weak physical interactions. These signals dissipated immediately upon transferring the sensor into the buffer, as illustrated in Figure 4b. If the A-DNAFL had bound to regions other than the Fc segment, a notable enhancement in signal should have been observed; however, no such signal was detected.

In the second verification experiment, the SAX2.0 sensor was functionalized with biotinylated A-DNAFL without prior antibody immobilization. Upon the addition of protein A, no measurable response signal was recorded (Figure 4c), consistent with the results shown in Figure 4a. In Figure 4d, following the immobilization of the antibody along with ssDNA, exposure to a 1 μM protein A solution yielded only a weak and transient signal, aligning with the findings in Figure 3b. Given that the nucleotide sequence may not fully encompass the Fc region, the limited interaction between protein A and the partially accessible epitope is expected. Consequently, only a weak binding signal, rather than a pronounced one, is observed throughout the experiment. Both competitive experiments provide strong evidence that the binding region of A-DNAFL to mouse IgG antibodies coincides with the recognition site of protein A, specifically the Fc segment. Therefore, the A-DNAFL proposed in this study holds promise as an alternative to protein A and could serve as a novel tool for the specific immobilization of mouse antibodies.

### 3.2. MDs Between ssDNA and IgG Antibody

**Figure 5:**
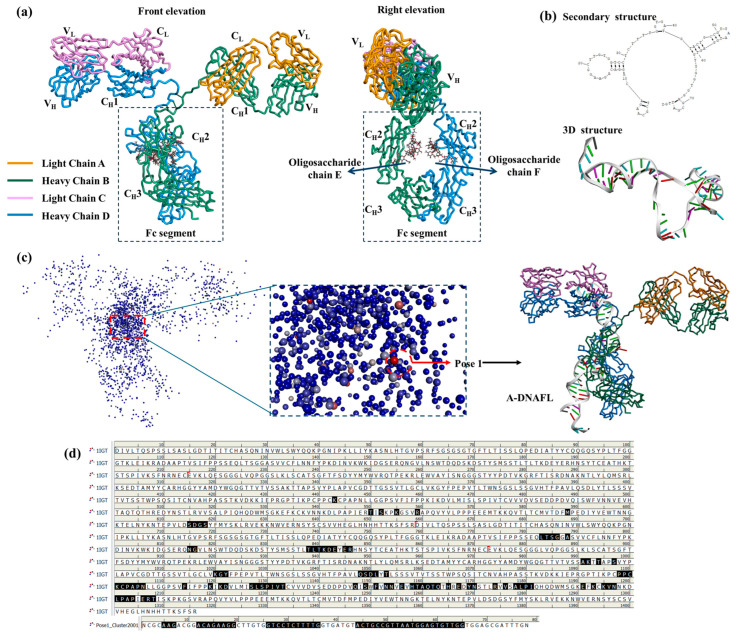
Molecular docking results of A-DNAFL with mouse IgG, (**a**) 3D structure of antibody (PDB ID: 1IGT); (**b**) secondary and 3D structures of the A-DNAFL; (**c**) distribution of 2000 potential poses and the most probable binding situation; (**d**) binding sites on the antibody amino acid sequence and nucleotide chain (The antibody protein sequence comprises 1316 amino acids, No.1 to No.214 residues correspond to light chain A, No.215 to No.658 residues constituting heavy chain B, No.659 to No.872 residues forming light chain C, and finally No.873 to No.1316 residues comprising heavy chain D. The boundaries between each chain are indicated by red markers, whereas the amino acids shown in black are situated at the interface between IgG and A—DNAFL).

PDB structure of the mouse IgG antibody, used as the receptor in the molecular docking simulation in this study, is illustrated in Figure 5a. As a classical IgG antibody, this structure comprises a complete light chain, heavy chain, and sugar chain, and exhibits a typical Y-shaped conformation. Meanwhile, secondary and 3D structures of the A-DNAFL molecule used as ligand are provided in Figure 5b. Docking result of the A-DNAFL with IgG is presented in Figure 5c. Following the completion of the molecular docking process using DS 4.5, the 2000 generated potential binding poses were ranked based on their ZDOCKScores. Conformation with the highest score, referred to as Pose 1, was selected as the most probable binding configuration, and A-DNAFL is theoretically bound to the Fc fragment of IgG obviously. In Figure 5d, the amino acid sequences of the antibody protein and the corresponding deoxyribonucleotide sequences are presented, with the interaction sites between the two sequences indicated in black. Secondary structure of A-DNAFL features a prominent left-side ring and an extended stem on the right, which serve as the primary sites involved in the binding process; meanwhile, it also predominantly interacts with the C_H_2 domain of the heavy chain D from the Fc fragment. As shown in Figure 5, A-DNAFL exhibits certain binding sites that even include the hinge region. Although such interactions are less favorable for directional immobilization compared to binding exclusively within the Fc fragment, they do not obstruct the full exposure of the antigen recognition domain, thereby preserving the functional integrity of the antibody without significant compromise.

**Figure 6:**
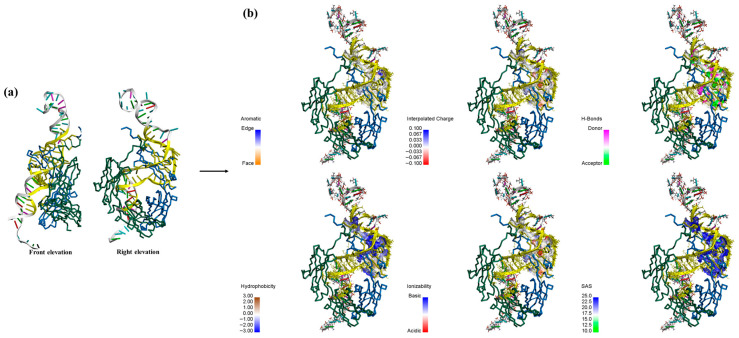
(**a**) zoom in on the Fc-A-DNAFL interface to view the sites and mark it in yellow; (**b**) interaction distribution at the receptor surface.

Figure 6a provides an enlarged view of the binding interface, and yellow highlights indicate the amino acid residues and nucleotide bases participating in the interaction. Furthermore, Figure 6b illustrates the distribution of six distinct types of interactions on the receptor surface, labeled as aromaticity, insertion charge, H-Bond, hydrophobicity, ionization, and solvent accessible surface (SAS), corresponding to the magnified view on the right side of Figure 6a. When the distance between any two atoms falls within 5 Å, the receptor and ligand residues are in close contact. In the aromaticity map, only a limited number of directional residue distributions are observed in the figure, including Trp290, Phe291, His302, His309, and Tyr313, and most of the surface appear white, signifying the absence of aromatic rings. Subsequently, most amino acid residues in the hydrophobicity map exhibit negative hydrophobicity values (depicted in blue), suggesting that the surrounding environment is predominantly hydrophilic. However, a limited number of highly hydrophobic residues, namely Leu268, Pro270, Ile271, and Val272, play a significant role in stabilizing the overall structural conformation. Furthermore, the interpolated charge distribution illustrates the proton-donating or proton-accepting capacity of amino acid residues located on the receptor surface. For instance, the ionized forms of Arg, His, and Lys carry positive charges, while Asp, Glu, and Tyr bear negative charges. In the H-Bond map, most of the binding surfaces are enriched with hydrogen bond donors and hydrogen bond acceptors, which include C=O groups as well as oxygen and nitrogen atoms along the molecular chain (illustrated in green). These features engage in interactions with the ssDNA through localized polarity, and the extensive H-Bond network plays a key role in enhancing the overall structural stability. SAS analysis enables the receptor surface exhibits a significantly larger solvent-exposed area (highlighted in blue as exposed residues), indicating a certain level of structural flexibility and functional plasticity, which may be associated with its relatively lower hydrophobicity. In summary, following the MDs of A-DNAFL with the IgG antibody, the receptor surface contains fewer aromatic and hydrophobic residues, which demonstrates a degree of flexibility and marked hydrophilicity, with the stability of the resulting complex primarily attributed to the presence of numerous H-Bond.

### 3.3. Toxin Detection

**Figure 7:**
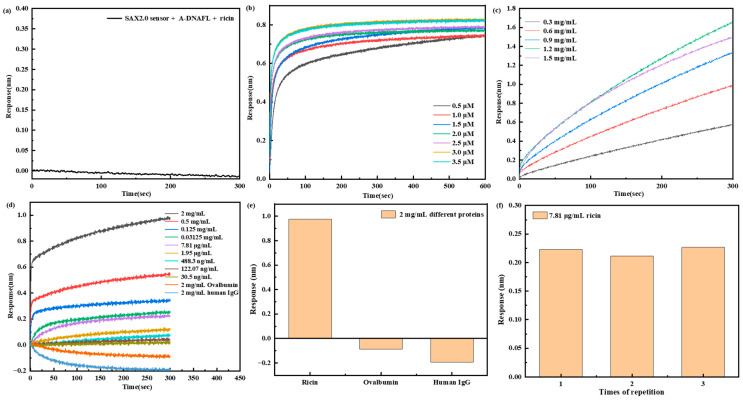
(**a**) Response of A-DNAFL to ricin; (**b**) signal of SAX2.0 sensor for saturating the immobilization of A-DNAFL; (**c**) signal of A-DNAFL saturating the immobilization of ricin McAb; (**d**) response of the sensors detect ricin and interfering substance; signal comparison of (**e**) different proteins and (**f**) reproducibility tests at a concentration of 7.81 μg/mL.

To develop a BLI-based immunosensor using A-DNAFL for the detection of ricin, which was selected as the immobilization matrix for the ricin McAb, based on previous experimental results. Initially, it was confirmed that the A-DNAFL immobilized on the SAX2.0 sensor did not produce any binding signal in the presence of ricin (Figure 7a). Then, Figure 7b illustrates the signal variation when the biotinylated A-DNAFL of SAX2.0 reaches saturation, and at an A-DNAFL concentration of 3 μM, the signal remains constant. Meanwhile, the maximum signal is observed at ricin McAb concentration of 1.2 mg/mL that can be presented in Figure 7c, which is considered the optimal immobilization concentration for the ricin McAb. Following the saturation, the sensor’s response to different concentrations of ricin was assessed, as shown in Figure 7d. Furthermore, the immunosensor was evaluated in solutions containing high concentrations of ovalbumin and human IgG. The signal responses were compared with those obtained for ricin toxin at the 2 mg/mL, as illustrated in Figure 7e. No detectable signals were observed for either protein, demonstrating the sensor’s high specificity and strong resistance to interference. Under identical experimental conditions, three replicate measurements were performed using a ricin toxin concentration of 7.81 μg/mL to assess repeatability. The average response signal was 0.202 nm, with a relative standard deviation (*RSD*) of 3.57%, demonstrating acceptable repeatability of the assay (Figure 7f).

**Figure 8:**
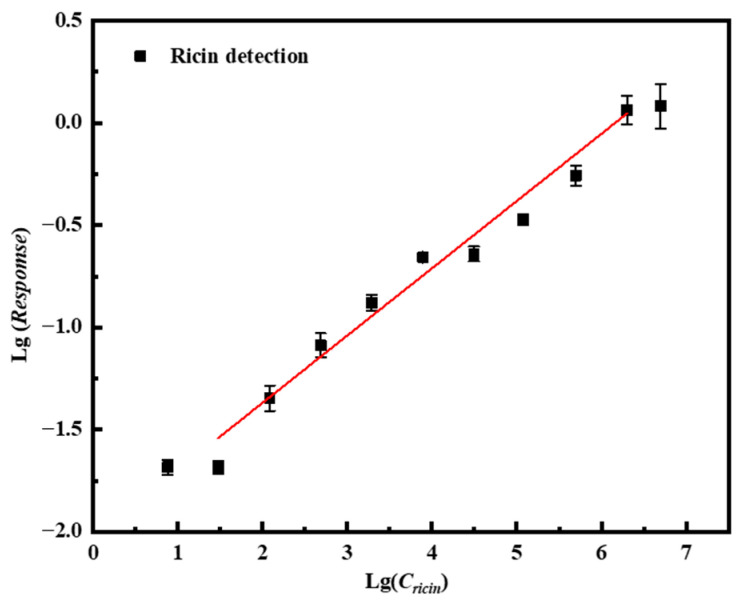
Linear curve of the immunosensor based on the A-DNAFL to the Fc segment for detecting ricin while the red line represents the fitted linear line curve.

When the concentration of ricin increased to 5 mg/mL or decreased to 7.625 ng/mL, the signal intensity plateaued without further increase or decrease, indicating that the andetection range of this immunosensor spans from 30.5 ng/mL to 2 × 10^6^ ng/mL. The limit of detection (LOD) was established as 30.5 ng/mL based on a signal-to-noise ratio of at least 3 (S/N ≥ 3). Each concentration was measured in triplicate, and the corresponding calibration curve along with error bars is presented in Figure 8. Within this dynamic range, the sensor response exhibits a logarithmic linear relationship with the ricin concentration, which can be expressed by the following linear regression equation: lg(*Response*) = 0.329 lg(*C_ricin_*) − 2.027 (*N* = 9, *R* = 0.938, *p* < 0.001).

Similarly, the results of detecting ricin using the ProA sensor with immobilized antibodies are presented in Figure S5. Initially, it was verified that protein A does not exhibit any response to ricin (Figure S5a), and the optimal antibody immobilization concentration was determined to be 1.2 mg/mL by analyzing the response signals obtained from different concentrations of ricin McAb bound to the ProA sensor (Figure S5b). Figure S5c illustrates the sensor’s response signals to varying concentrations of ricin, yielding a detection limit of 1.95 μg/mL (S/N ≥ 3). As shown in Figure S5d, the sensor was subjected to three replicate measurements of ricin at a concentration of 62.5 μg/mL. and the resulting response signal was 0.076 nm, corresponding to *RSD* of 17.13%. The linear curve fitting the concentration to the signal value is presented in Figure S5e, with the corresponding equation expressed as lg(*Response*) = 0.158 lg(*C_ricin_*) − 2.003 (*N* = 5, *R* = 0.781, *p* < 0.001). Therefore, under the same BLI detection interface conditions, a comparison of the two sensor configurations demonstrates that the detection limit achieved using antibody immobilization based on A-DNAFL is 64 times lower than that achieved using protein A-based immobilization.

## 4. Discussion

Therefore, based on these research findings, the A-DNAFL obtained from previous studies conducted by the research group can serve as an effective antibody-immobilization material. It demonstrates strong binding affinity toward the Fc fragment of mouse IgG antibodies and can be readily modified onto sensing interfaces for the specific immobilization of IgG antibodies of this species. Moreover, this highly compatible single-stranded DNA exhibits low molecular weight, simple preparation and easy modification, which is advantageous for biosensor design. In this study, an immunosensor based on BLI technology was successfully developed, achieving a detection limit 64 times lower than that of the conventional protein A-based immobilization method, which results suggest that A-DNAFL holds significant potential for applications in the field of biosensing.

**Figure 9:**
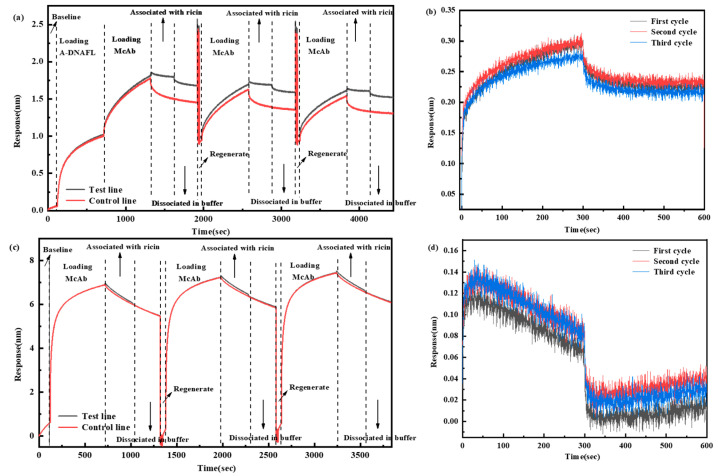
Results of repeated toxin detection using the two types of sensors after regeneration, (**a**) full process of detecting after immobilizing antibodies by A-DNAFL; (**b**) ternary cycle after subtracting the reference curve from (**a**); (**c**) full process of detecting after immobilizing antibodies by protein A; (**d**) ternary cycle after subtracting the reference curve from (**c**). (The dashed lines in the figure are used to distinguish different stages).

However, antigen–antibody recognition based immunosensors face significant challenges in practical applications, including high antibody production costs, complex preparation procedures, and limited reusability. To overcome these limitations, this study further explores the regeneration capability of the BLI immunosensing interface utilizing A-DNAFL. The two BLI sensors were immediately dissociated after detecting the same concentration (0.125 mg/mL) of the ricin, followed by regeneration using glycine to remove the antibodies bound to either A-DNAFL or protein A, and regeneration process was repeated three times. The complete signal curves are illustrated in Figure 9a,c, while the detection signals from the three cycles, after subtracting the reference signal, are presented in Figure 9b,d. The signal value of the A-DNAFL-immobilized antibody remained stable after the first regeneration cycle but decreased from 0.300 to 0.274 nm after the second regeneration, representing a reduction of 8.55%. In contrast, the signal value of the antibody immobilized via protein A decreased significantly, with the overall response dropping below 0.1 nm within just 5 min of binding. Therefore, in the BLI system, A-DNAFL demonstrates superior performance as an antibody immobilization material. However, if the affinity of A-DNAFL to the antibody is one order of magnitude higher than that of the antigen–antibody complex, it becomes feasible to completely dissociate the antigen without compromising the integrity of the A-DNAFL-antibody complex, thereby enabling efficient recovery of the antibody and facilitating reuse of the immunosensing interface.

## 5. Conclusions

In this study, we leveraged the findings from our previous work—A-DNAFL—to investigate the binding affinity between specific single-stranded DNA sequences and classical mouse IgG antibodies, which KD is 6.59 × 10^−8^. We demonstrated that A-DNAFL can specifically recognize these antibodies and identified the optimal binding PBST-B conditions (pH 7.2–7.4, containing approximately 154 mM Na^+^ and 4 mM K^+^) by systematically varying pH and ionic strength across different buffer systems. Through two protein A-based competitive binding assays, we determined that the A-DNAFL binds specifically to the Fc fragment of the IgG antibody. Subsequently, MDs were performed to elucidate the theoretical binding mode, and results indicated that the C_H_2 region of the heavy chain D, along with the large ring on the left side and the stem structure on the right side of the DNA secondary structure, constituted the primary binding sites. The interaction interface is predominantly stabilized by hydrogen bonds, with minimal contribution from hydrophobic interactions. Leveraging this A-DNAFL as a molecular anchor for IgG immobilization, we developed an immunosensor based on BLI technology. The linear regression equation of this biosensor is lg(*Response*) = 0.329 lg(*C_ricin_*) − 2.027 (*N* = 9, *R* = 0.938, *p* < 0.001), which exhibited a detection limit of 30.5 ng/mL, representing a 64-fold improvement in sensitivity compared to conventional protein A-based immobilization methods. Furthermore, it demonstrated excellent reproducibility, a broad linear detection range, and robust resistance to interference. Furthermore, the regeneration experiments demonstrated that the BLI immunosensing interface based on A-DNAFL could be effectively regenerated through glycine washing, enabling reuses and thereby achieving significant cost savings. A-DNAFL targeting the Fc fragment of IgG antibodies, as proposed in this work, represents a novel antibody immobilization tool that offers a viable alternative to conventional protein A, which has a higher molecular weight and expensive costs. This approach not only facilitates the construction of highly sensitive immunosensors but also supports repeated antibody reuse, thereby significantly reducing experimental costs. Given these advantages, the developed material holds considerable promise for broad applications in life sciences, environmental monitoring, and clinical diagnostics.

## Data Availability

The original contributions presented in this study are included in the article/Supplementary Materials. Further inquiries can be directed to the corresponding author.

## Supplementary Materials

The following supporting information can be downloaded at: https://www.mdpi.com/article/10.3390/bios15110747/s1, Figure S1: Two competitive experiments for verifying the binding sites; Figure S2: Process of detecting ricin, (a) the novel immunosensor based on A-DNAFL; (b) traditional immunosensor based on protein A; Figure S3: Association-dissociation curves of A-DNAFL and IgG antibody under different pH conditions, (a) pH 5.0; (b) pH 6.0; (c) pH 7.0; (d) pH 8.0; (e) pH 9.0; Figure S4: Associaiton-dissociation curves of A-DNAFL and IgG antibody under varying ionic strengths, (a) 10x K^+^; (b) 10x Na^+^; (c) 0.1 mM Mg^2+^; (d) 1 mM Mg^2+^; (e) 10 mM Mg2+; Figure S5: (a) Response of protein A to ricin; (b) signal of ProA sensor for saturating the immobilization of ricin McAb; (c) detection for concentrations of ricin; (d) response of three reproducibility tests at a concentration of 62.5 μg/mL; (e) linear curve of the immunosensor based on the protein A for detecting ricin.

## Abbreviations

The following abbreviations are used in this manuscript:

A-DNAFL	Affinity DNA functional ligand
Fab	Antigen-binding fragments
Fc	Crystallizable fragment
BLI	Bio-Layer Interferometry
Ricin McAb	Mouse anti-ricin monoclonal antibody
BSA	Bovine serum albumin
SAX2.0	High Precision Streptavidin
ProA	Protein A
DS4.5	Discovery Studio 4.5
MDs	Molecular docking simulations
SAS	Solvent accessible surface
Human IgG	Human immunoglobulin G
RSD	Relative standard deviation
