# Supplementary material for: Construction of an Immunosensor Based on the Affinity DNA Functional Ligands to the Fc Segment of IgG Antibody
Source: Biosensors (Basel). 2025 Nov 5;15(11):747. doi: 10.3390/bios15110747 (PMC12649849; doi:10.3390/bios15110747)
Supplement: Supplementary file 1 [file biosensors-15-00747-s001.zip › biosensors-3932879-supplementary.pdf]

Construction of an immunosensor based on the affinity DNA functional ligands to the Fc segment of IgG antibody

Qianyu Yang, Zhiwei Liu*. Xinrui Xu, Zihao Zhao, Ze Fan, Bin Du, Jianjie Xu, Jiwei Xu, Jiang Wang, Bing Liu, Xihui Mu, Zhaoyang Tong*

State Key Laboratory of Chemistry for NBC Hazards Protection, Beijing 102205.

*: Corresponding author: Zhaoyang Tong (billzytong@126.com); Zhiwei Liu (liuzhw07@lzu.edu.cn)

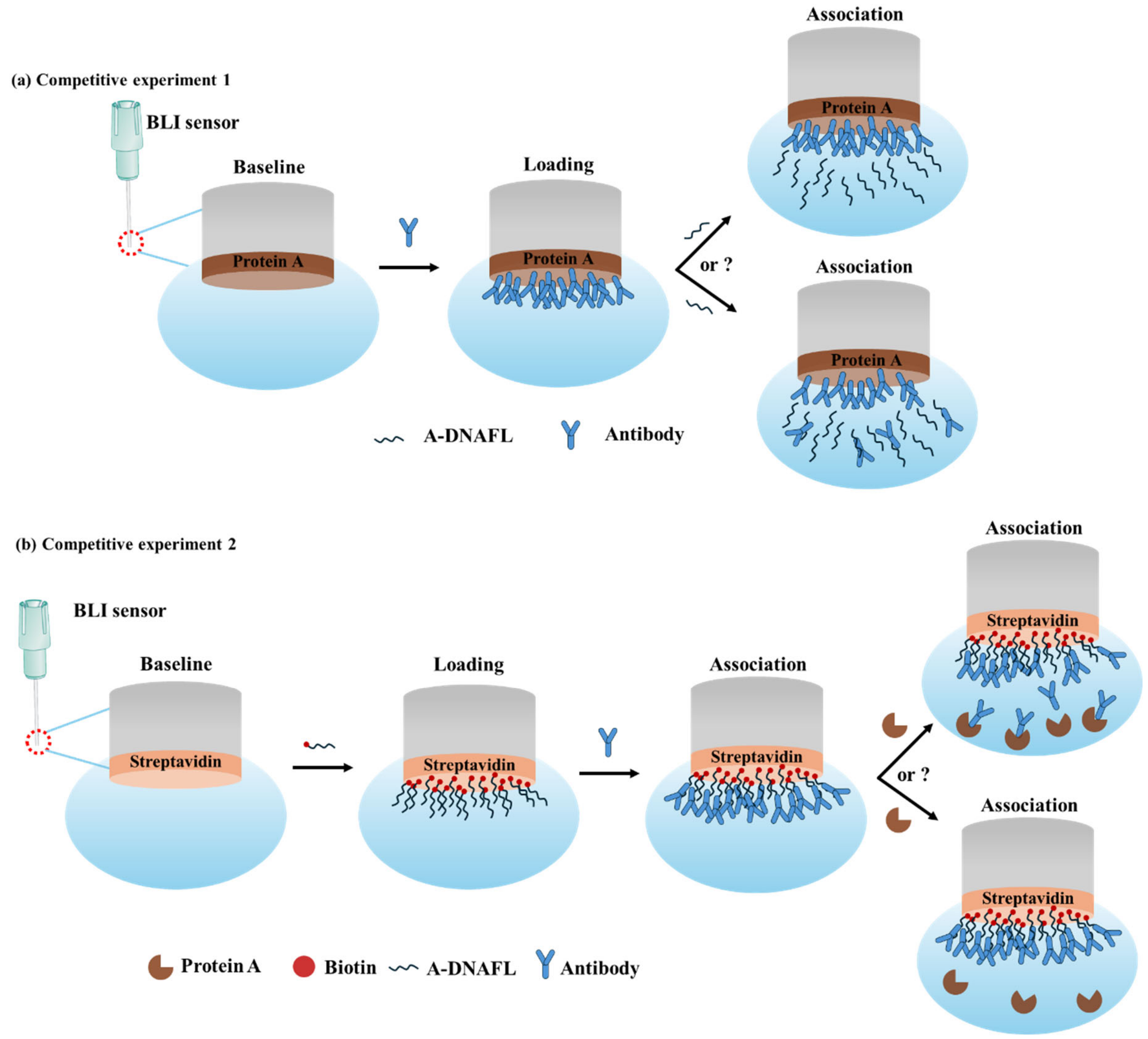

Figure S1. Two competitive experiments for verifying the binding sites.

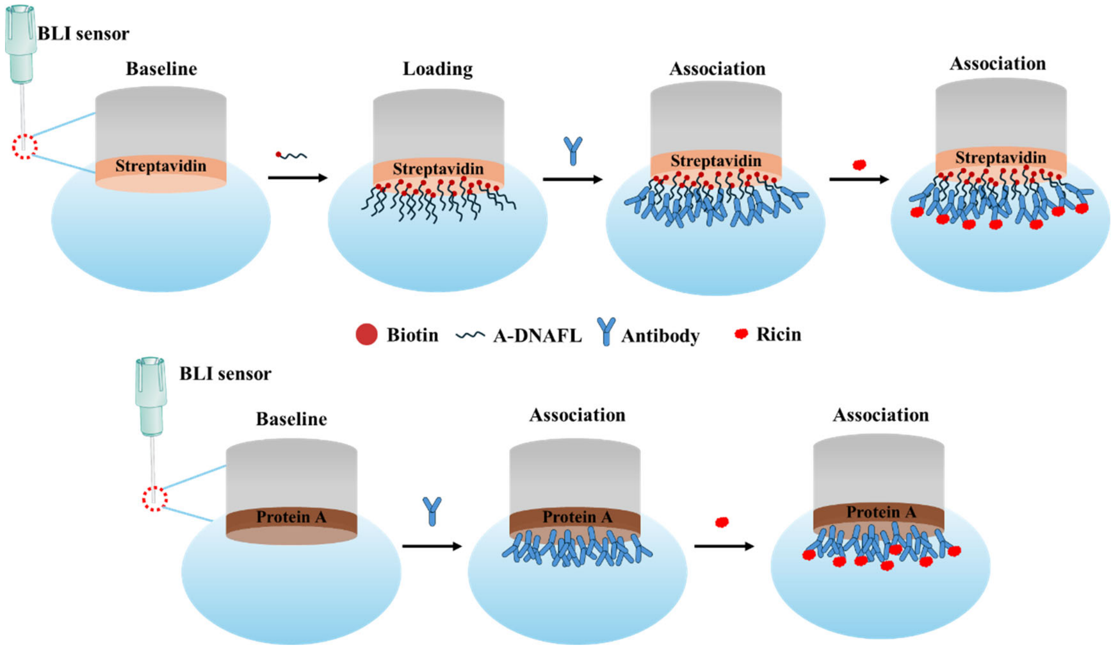

Figure S2 Process of detecting ricin, (a) the novel immunosensor based on A-DNAFL; (b) traditional immunosensor based on protein A.

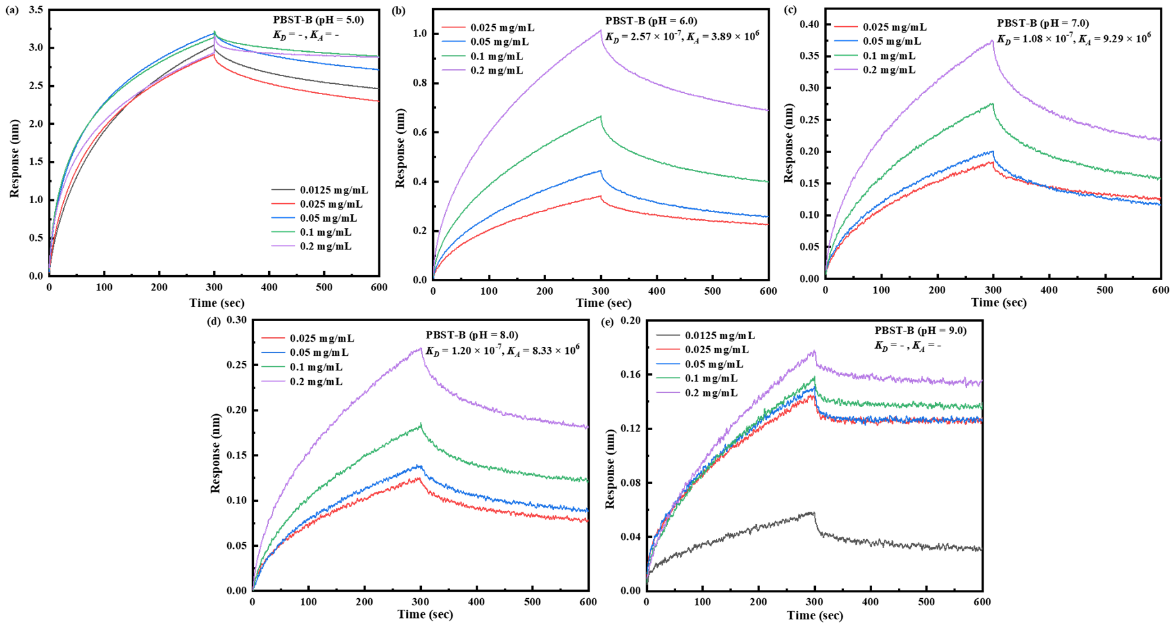

Figure S3 Associaiton-dissociation curves of A-DNAFL and IgG antibody under different pH conditions, (a) pH 5.0; (b) pH 6.0; (c) pH 7.0; (d) pH 8.0; (e) pH 9.0.

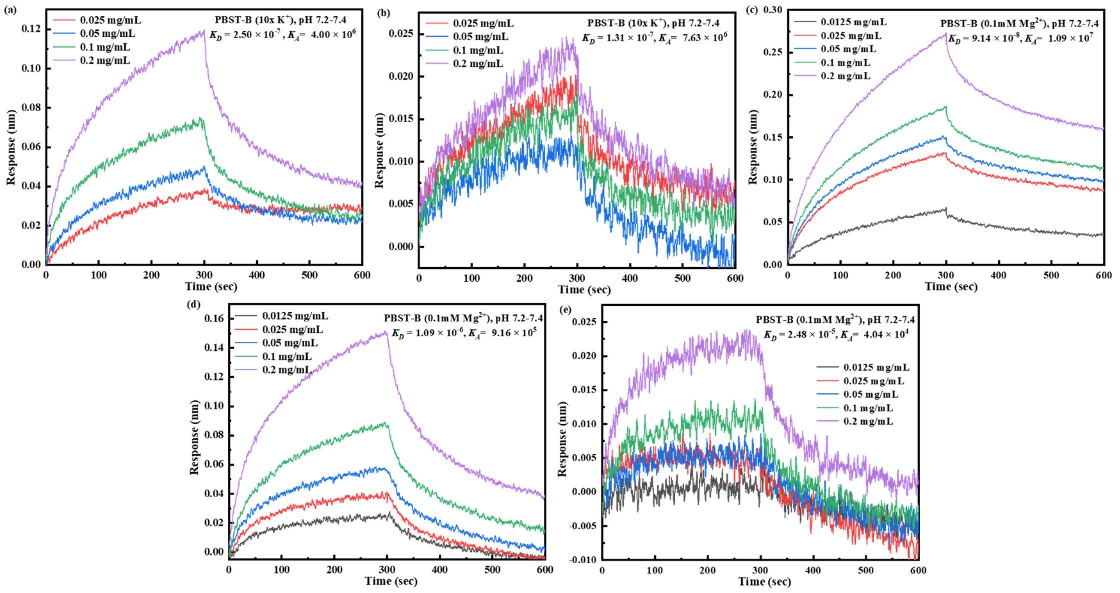

Figure S4 Association-dissociation curves of A-DNAFL and IgG antibody under varying ionic strengths, (a) 10x K^+; (b) 10x Na^+; (c) 0.1 mM Mg^{2+}; (d) 1 mM Mg^{2+}; (e) 10 mM Mg^{2+}.

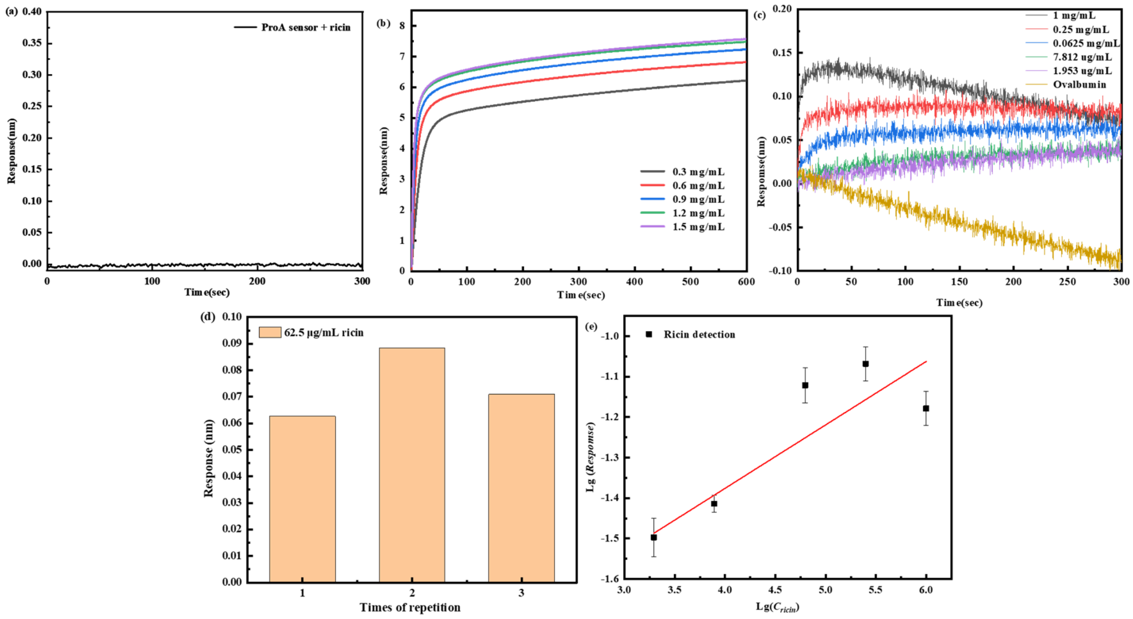

Figure S5 (a) Response of protein A to ricin; (b) signal of ProA sensor for saturating the immobilization of ricin McAb; (c) detection for concentrations of ricin; (d) response of three reproducibility tests at a concentration of 62.5 μg/mL; (e) linear curve of the immunosensor based on the protein A for detecting ricin.
